# A Retrospective Study of G-Tube Use in Japanese Patients Treated with Concurrent Chemoradiotherapy for Hypopharyngeal Cancer

**DOI:** 10.1371/journal.pone.0161734

**Published:** 2016-08-24

**Authors:** Akihiro Homma, Hiromitsu Hatakeyama, Takatsugu Mizumachi, Satoshi Kano, Tomohiro Sakashita, Rinnosuke Kuramoto, Yuji Nakamaru, Rikiya Onimaru, Kazuhiko Tsuchiya, Daisuke Yoshida, Koichi Yasuda, Hiroki Shirato, Satoshi Fukuda

**Affiliations:** 1 Department of Otolaryngology-Head & Neck Surgery, Hokkaido University Graduate School of Medicine, Sapporo, Japan; 2 Department of Radiation Medicine, Hokkaido University Graduate School of Medicine, Sapporo, Japan; University of South Alabama Mitchell Cancer Institute, UNITED STATES

## Abstract

**Objective:**

Late toxicity after concurrent chemoradiotherapy (CCRT), such as dysphagia, in patients with squamous cell carcinoma of the head and neck has received a good deal of attention recently. The gastrostomy tube (G-tube) dependence rate 1 year after CCRT was reported to be 16.7–42.9% in Western countries. We evaluated swallowing outcomes after CCRT in patients with hypopharyngeal cancer (HPC) treated in our hospital and compared them with previous reports.

**Methods:**

We reviewed 96 consecutive patients with a HPC treated by radiotherapy with intravenous or intra-arterial chemotherapy between 2006 and 2013 at Hokkaido University Hospital, Sapporo, Japan.

**Results:**

At 1 month after CCRT, 13 patients (13.7%) used a G-tube, whereas 5/91 (5.5%) and 4/81 (4.9%) used a G-tube at 3 and 6 months, respectively. Two patients used a G-tube at 12 and 24 months after CCRT (G-tube use rate: 2.8% at 12 months, and 3.2% at 24 months). The variables female, posterior wall primary, stage IV, ECOG performance status of 2, and smoking status were significantly associated with G-tube use at 12 months after CCRT, whereas the route of cisplatin administration was not related to G-tube use (p = 0.303).

**Conclusions:**

The G-tube use rate up to 1year could be lower in Japanese patients than in Western patients according to previous reports. In particular, Japanese patients resume oral intake sooner than Western patients. Further study of the incidence of dysphagia after CCRT by ethnicity is required to clarify the differences in dysphagia after CCRT.

## Introduction

Concurrent chemoradiotherapy (CCRT) is a standard treatment of the care for patients with locally advanced squamous cell carcinoma of the head and neck when treated nonsurgically. However, late toxicity after CCRT, such as dysphagia; i.e., difficulty swallowing and the need for tube feeding or parenteral nutrition, has received a good deal of attention recently. Caudell *et al*. reported that 38.5% of patients with locoregionally advanced head and neck cancer treated with definitive radiotherapy had late severe dysphagia [1]. Although radiation doses to the larynx and pharyngeal constrictors have been reported to be strongly associated with swallowing outcomes [2], such structures are generally the primary target and cannot be spared in patients with hypopharyngeal cancer (HPC), even when advanced irradiation techniques, such as intensity-modulated radiotherapy (IMRT), are employed [3]. Therefore, patients with HPC are considered to be more likely to develop dysphagia after CCRT than those with cancer located at other sites in the head and neck in Western countries. Bhayani *et al*. reported that 11 (25.6%) of 43 patients with HPC who had a complete response at the primary site after radiotherapy with/without chemotherapy remained dependent on a gastrostomy tube (G-tube) at 1 year post-treatment [4]. Paximadis *et al*. also reported that 8 (16.3%) of 49 patients with HPC treated by radiotherapy with/without chemotherapy required a permanent G-tube, with a median follow-up period of 18 months [5]. On the other hand, patients with dysphagia after CCRT are not often encountered in a daily practice in Japan. Therefore, we evaluated swallowing outcomes after CCRT in patients with HPC treated in our hospital and compared the results with those from previous reports.

## Patients and Methods

We retrospectively reviewed the records of 96 consecutive patients with a HPC of squamous cell carcinoma treated by radiotherapy with intravenous (IV) or intra-arterial (IA) chemotherapy between 2006 and 2013 at Hokkaido University Hospital, Sapporo, Japan.

Seventy-five patients were treated by radiotherapy with IV cisplatin and 21 with IA cisplatin. The former consisted of weekly cisplatin (40 mg/m^2^) given intravenously on weeks 1, 2, 3, 5, 6 and 7 [6], and the latter consisted of superselective intra-arterial infusions of cisplatin (100-120mg/m^2^ per week) with simultaneous intra-venous infusions of thiosulfate to neutralize cisplatin toxicity [7,8]. Indications for IA-CCRT were basically defined as unilateral primary tumors staged as T3-4a and N0-1. However, patients with poor renal function, such as a creatinine clearance of approximately 50 to 60 mL/min, were more likely to be recommended IA-CCRT because we consider that it affords better compliance with cisplatin administration than IV-CCRT. In addition, IA-CCRT was indicated for patients who preferred IA-CCRT to IV-CCRT.

Patients were treated by 3-dimensional conformal radiotherapy (3DCRT) until April 2013, and thereafter all patients were treated by intensity-modulated radiotherapy (IMRT). For both methods, a standard dose of 70 Gy was delivered in 35 daily fractions over 7 weeks. The initial plan of 44–46 Gy/22-23 fractions included the primary site, metastatic lymph nodes and regional lymphatic area from the retropharyngeal nodes to the supraclavicular fossa. The boost plan of 24–26 Gy/12-13 fractions was made to the primary site and metastatic lymph nodes.

Patients in particularly good shape with N2c-3 and/or Level IV or V lymph node metastasis received three cycles of induction chemotherapy (docetaxel 75 mg/m^2^ and cisplatin 75 mg/m^2^, day 1; and 5-fluorouracil 750 mg/m^2^/day 120 h continuous infusion, every 3 weeks) followed by IV- or IA-CCRT [9].

All patients were basically recommended G-tube placement before or at the early stage of treatment in case they could not have oral intake later in the treatment period. Patients who were able to receive adequate oral intake after treatment naturally dispensed with G-tube use and the G-tube was removed. Active swallowing exercises were not introduced during this study period.

The data on swallowing status were gathered from patients' interviews in their medical records at baseline, and 1, 3, 6, 12, and 24 months after CCRT. Smoking status was stratified as patients who never smoked (never), those who quit smoking at any time prior to diagnosis (former), or those who smoked at the time of diagnosis (current).

Approval for this study was obtained from the Institutional Review Board at Hokkaido University and patient records/information was anonymized and de-identified prior to analysis.

### Statistical analysis

All patients were observed closely during follow-up. The median follow-up period for surviving patients was 5.2 years (average 5.3 years, range 2.2–9.7 years).

Patients who required a feeding tube or parenteral nutrition were defined as “G-tube use”. G-tube use rate was analyzed in patients surviving without primary site recurrence at 1, 3, 6, 12 and 24 months after therapy. Contingency table analyses based on the unpaired Student’s t- test or the chi-square test were used to determine the statistical significance of associations between categorical variables. Probabilities of overall survival, which included death from any cause computed from the beginning of treatment to the time of death, were calculated by the Kaplan-Meier method. The level of statistical significance was defined as a 2-tailed p < .05. Statistical analysis was performed using JMP Pro 12.0.1 statistical software (SAS Institute, Cary, NC).

## Results

### Patient and treatment characteristics

Patient characteristics are summarized in Table 1. The median age of patients at diagnosis was 61 years (mean 60.6 years, range, 45–75 years), and 89 (92.7%) of 96 patients were male. T classifications were as follow: T1 (n = 3), T2 (48), T3 (18), T4a (21), and T4b (6). Lymph node involvement was noted in 76 patients (79.2%). Nine patients (9.4%) had dysphagia at diagnosis.

**Table 1:** Patient characteristics.

Characteristics		Total
Age (range, 45–75; median 61)		
	< 60	44
	≥ 60	52
Sex		
	Male	89
	Female	7
Subsite		
	Pyriform sinus	88
	Posterior wall	8
T classification		
	1	3
	2	48
	3	18
	4a	21
	4b	6
N classification		
	0	20
	1	14
	2a	1
	2b	39
	2c	14
	3	8
Stage		
	II	13
	III	16
	IVA	55
	IVB	11
	IVC	1
Performance status (ECOG)		
	0	53
	1	36
	2	7
Smoking		
	Current	62
	Former	24
	Never	10
Baseline dysphagia		
	Yes	9
	No	87

Seventy-five patients received IV-CCRT and 21 received IA-CCRT (Table 2). Fourteen patients (14.6%) received IMRT and the remaining 82 patients (85.4%) received 3DCRT. Induction chemotherapy was indicated for 23 patients (24%). G-tubes were placed in 46 patients (47.9%). Fourteen patients had a G-tube placed before the start of CCRT, 30 early during CCRT, and 2 after CCRT. Fifty patients did not undergo G-tube placement: 9 for medical reasons and 41 due to the patients’ wishes. The latter indicated that they did not want a G-tube to be placed in advance. They received either a nasogastric tube or parenteral nutrition when transoral intake was insufficient.

**Table 2:** Treatment details and G-tube placement.

Characteristics	Total
Chemotherapy	
IV cisplatin	75
IA cisplatin	21
Radiotherapy	
IMRT	14
3D-CRT	82
Induction chemotherapy	
Yes	23
No	73
G-tube placed	
Yes	46
No	50
Timing of G-tube placement	
before CCRT	14
during CCRT	30
after CCRT	2

CCRT: concurrent chemoradiotherapy, G-tube: gastrostomy tube

**Fig 1:**
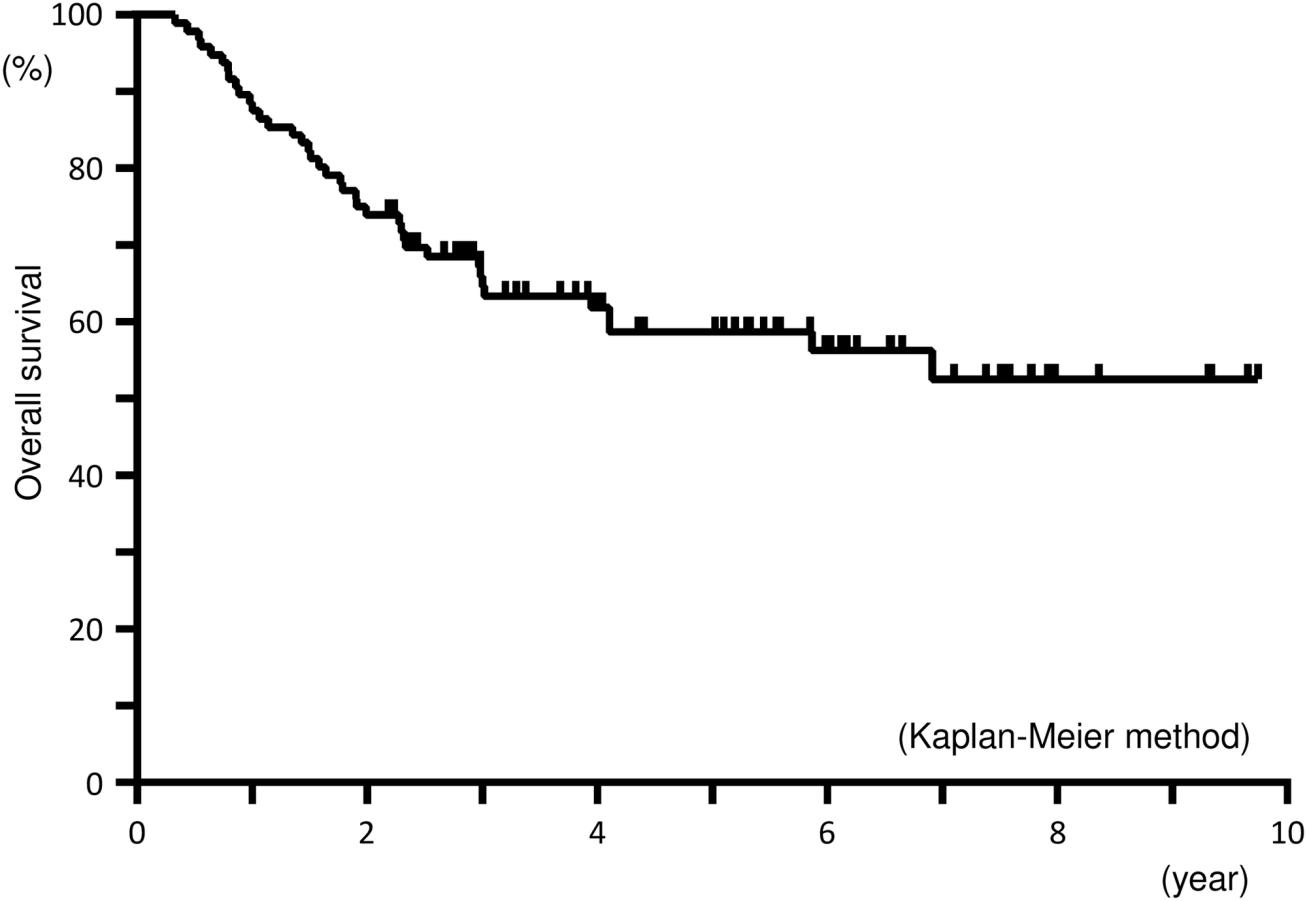
Overall survival for all patients. The 2-year and 5-year overall survival rates were 74.0% and 58.7%, respectively.

The 2-year and 5-year overall survival rates for all patients were 74.0% (95% confidence interval [CI]: 64.3%-81.8%), and 58.7% (95% CI: 48.0%-68.7%), respectively (Fig 1).

### Swallowing outcomes

Nine patients were G-tube use at baseline. Table 3 details swallowing outcomes at 1, 3, 6, 12, 24 months after CCRT. One patient could not receive oral intake due to pharyngeal stricture after IV-CCRT and subsequently underwent total laryngo-pharyngectomy and reconstruction by free jejunum flap. He was able to eat anything transorally after surgery, but was treated as “tube feed only” in this analysis even after surgery. At 1 month after CCRT, 13 patients (13.7%) used a G-tube, and 5/91 (5.5%) and 4/81 (4.9%) continued to use a G-tube at 3 and 6 months, respectively. One patient who used G-tube at baseline has not been able to eat transorally to date; i.e., during and after IA-CCRT. Only 2 patients mentioned above continued to use a G-tube at 12 and 24 months after CCRT (G-tube use rate: 2.8% at 12 months, and 3.2% at 24 months).

**Table 3:** Swallowing outcomes.

Follow-up duration (months)		Tube feed only		Tube + oral intake		Oral intake only	
	No. of patients	No. of patients	%	No. of patients	%	No. of patients	%
baseline	96	3	3.1%	6	6.3%	87	90.6%
1	95	6	6.3%	7	7.4%	82	86.3%
3	91	4	4.4%	1	1.1%	86	94.5%
6	81	4	4.9%			77	95.1%
12	72	2	2.8%			70	97.2%
24	63	2	3.2%			61	96.8%

Table 4 shows the relationships among G-tube use at 12 months after CCRT and patient characteristics and treatments. The variables female, posterior wall primary, ECOG performance status of 2, baseline G-tube use, and smoking status were significantly associated with G-tube use at 12 months, whereas the route of cisplatin administration was not related to G-tube use (p = 0.303).

**Table 4:** Factors associated with G-tube use at 12 months after CCRT.

Clinical variables		G-tube use		Total	*p*
		(-)	(+)		
Age					
	< 60	33	1	34	
	≥ 60	37	1	38	0.936
Sex					
	Male	66	1	67	
	Female	4	1	5	0.015
Subsite					
	Pyriform sinus	68	1	69	
	Posterior wall	2	1	3	0.001
T classification					
	T1-2	45	0	45	
	T3-4	25	2	27	0.064
N classification					
	N-	16	1	17	
	N+	54	1	55	0.373
Stage					
	II-III	22	1	23	
	IV	48	1	49	0.579
Radiotherapy					
	IMRT	11	0	11	
	3DCRT	59	2	61	0.543
Performance status (ECOG)					
	0–1	67	1	68	
	2	3	1	4	0.005
Baseline G-tube use					
	Yes	3	1	4	
	No	67	1	68	0.005
G-tube placed (by the end of CCRT)					
	Yes	29	1	30	
	No	41	1	42	0.808
Smoking					
	current	49	0	49	
	former	17	0	17	
	never	4	2	6	< .0001
Treatment					
	IV-CCRT	56	1	57	
	IA-CCRT	14	1	15	0.303
Induction chemotherapy					
	Yes	12	1	13	
	No	58	1	59	0.234
Weight loss rate					
	≥ 10%	21	0	21	
	< 10%	49	2	51	0.357

IMRT: intensity-modulated radiotherapy, 3DCRT: 3-dimensional conformal radiotherapy, CCRT: concurrent chemoradiotherapy, G-tube: gastrostomy tube

## Discussion

Dysphagia is one of the most important toxicities after CCRT for patients with head and neck cancer as patients receive CCRT in the hope that they can speak and swallow after therapy. HPC is considered to be more likely to lead to dysphagia than other head and neck cancers, such as laryngeal and oropharyngeal cancer [10]. Therefore, accurate data regarding toxicity after CCRT is needed to allow patients with HPC to be informed in advance and not prevent the use of CCRT due to excessive concern about treatment toxicity [11].

In this study, 13 of 95 patients who continued follow-up without residual/recurrent tumor (13.7%) used a G-tube at 1 month after CCRT, and 5/91 (5.5%), 4/81 (4.9%), and 2/72 (2.8%) continued to use a G-tube at 3, 6, and 12 months, respectively. This result was lower than the figures in previous reports from Western countries (Table 5). Ackerstaff *et al*., from The Netherlands, assessed the quality of life of patients after IA- versus IV-CCRT for inoperable stage IV head and neck cancer [12]. Tube feeding rates were 79/88 (89.8%) at 7 weeks after treatment, 58/88 (65.9%) at 3 months, and 10/60 (16.7%) at 12 months in the IA group, and 78/95 (82.1%) at 7 weeks, 64/92 (69.6%) at 3 months, and 16/66 (24.2%) at 12 months in the IV group. However, around 30% of patients enrolled in the Dutch study required tube feeding at baseline as patients with inoperable stage IV disease were eligible. Tsao *et al*., from the MD Anderson Cancer Center, reported that the rates of G-tubes in place were 76.9%, 72.5%, 56%, and 42.9% at 6 weeks, and 3, 6, and 12 months, respectively, after IV-CCRT among patients with stage III-IV head and neck cancer [13]. On the contrary, Inohara *et al*., from Japan, reported that the rates of G-tubes in place were 12.6%, 8.9%, 5.6%, and 7.1% at 6 weeks, and 3, 6, and 12 months, respectively after IV-CCRT among patients with stage III-IV head and neck cancer [14]. Further, Wakisaka *et al*., also from Japan, reported that the tube feeding rate at 6 months after IV- or IA-CCRT was 16%. Taken together with the results of our study, these findings suggest that the G-tube use rate up to 12 months is lower in Japanese patients than in Western patients.

**Table 5:** Dysphagia after CCRT.

Author (country, year)	Dysphagia				Definition of dysphagia or Dysphagia rate
Patients					
Treatment modality	3 m	6 m	12 m	24 m	
Tsao [13] (USA, 1999–2002)	72.5%	56.0%	42.9%	34.3%	G-tube in place
n = 52, Stage III-IV HNSCC	(37/51)	(28/50)	(18/42)	(12/35)	6w:76.9%(40/52)
CCRT (cisplatin+docetaxel, concomitant boost)					
Ackerstaff [12] (Netherlands, 1999–2004)	65.9%		16.7%		need for tube feeding
n = 104, inoperable stage IV	(58/88)		(10/60)		7w: 89.8%(79/88), 5y: 2.8%(1/36)
CCRT(IV-cisplatin)					
Ackerstaff [12] (Netherlands, 1999–2004)	69.6%		24.2%		need for tube feeding
n = 103, inoperable stage IV	(64/92)		(16/66)		7w: 82.1%(78/95), 5y: 17.1%(6/35)
CCRT(IA-cisplatin)					
Garden [26] (RTOG 9914, USA, 2000)			40.9%	21.8%	prevalence of G-tube
n = 76, Stage III-IV HNSCC(HPC:8)					3y: 18.1%, 4y: 16.7%
CCRT (cisplatin, concomitant boost)					
Shiley [27] (USA, 1994–2003)	66.70%	50%			need for tube feeding
n = 30, stage III-IV OPC	(18/27)	(12/24)			last follow up: 51.8% (14/27)
IC-RT:8, IC-CCRT:4, CCRT:15					
Bhayani [18] (USA, 2003–2008)		22.2%	20.8%	14.8%	maintain feeding tubes
n = 474, OPC		(107/470)	(41/464)	(17/427)	
RT:115, IC-RT:73, CCRT:218, IC-CCRT:69					
Bhayani [4] (USA, 2002–2008)			25.6%	3.4%	maintain feeding tubes
n = 56 HPC			(11/43)	(1/29)	
RT:2, IC-RT:2, CCRT:25, IC-CCRT:14					
Murono [23] (Japan, 2002–2012)		16%			need for tube feeding
n = 75 HPC		(12/75)			
CCRT (IV:35, IA:40)					
Inohara [14] (Japan, 2004–2011)	12.6%	8.9%	7.1%	7.8%	G-tube in place
n = 116, Stage III-IV resectable HNSCC(HPC:54)	(13/103)	(9/101)	(6/85)	(6/77)	
CCRT(cislatin+docetaxel)					
This study (Japan, 2006–2013)	5.5%	4.9%	2.8%	3.2%	G-tube use
n = 96 HPC	(5/91)	(4/81)	(2/72)	(2/63)	1m: 13.7%(13/95)
CCRT(IV cisplatin:75, IA cisplatin:21)					

CCRT: concurrent chemoradiotherapy, HNSCC: head and neck squamous cell carcinoma, G-tube: gastrostomy tube, IC: induction chemotherapy, RT: radiotherapy, OPC: oropharyngeal cancer, HPC: hypopharyngeal cancer

As for G-tube dependence at 2 or more years after CCRT, Lee *et al*., from the Memorial Sloan Kettering Cancer Center, reported that Kaplan-Meier estimate of the G-tube dependence rate for 11 patients with HPC after IMRT with chemotherapy was 31% after 2 years [3]. And a landmark phase III study on HPC conducted by the European Organization for Research and Treatment of Cancer reported that 5 patients (9.6%) had a feeding tube or a gastrostomy during the follow-up period among 52 patients receiving induction chemotherapy followed by radiotherapy with laryngeal preservation [15]. In this study, 2 patients continued to use a G-tube at 12 and 24 months after CCRT (G-tube use rate: 2.8% at 12 months, and 3.2% at 24 months). Looking at reports from South Korea, Jang *et al*., reported that severe dysphagia requiring alternative feeding occurred in 14 (13.1%) of 107 patients with stage III-IVA HPC [16]. Yoon reported that 19 patients retained their larynx for more than 3 years among 66 patients with stage III-IV HPC treated by CCRT or induction chemotherapy followed by radiotherapy, and none required a feeding tube or a gastrostomy [17]. Reports from Korea and Japan appear to show lower G-tube dependence after 2 or more years than do Western reports. However, a recent report from the MD Anderson Cancer Center described the introduction of an aggressive targeted swallowing exercise regimen, with only one (3.4%) of 29 patients with HPC who continued follow up remaining dependent on a feeding tube at 2 [18]. G-tube dependence rates appear to fall over time in the reports from Western countries, although they are already low in the early stages after CRT in Japanese reports (Table 5). However, there might not have been any difference in the G-tube dependence rate at 2 years after CCRT between this study and previous reports.

This study suggests that our patients resume oral intake sooner than do Western patients. Western patients mostly resume oral intake at 2 or more years after treatment. One explanation for why Japanese patients are less likely to develop dysphagia early after CCRT is as follows. First, the pharyngeal constrictor muscles, including the cricopharyngeal muscle, are considered to have an important role in the development of pharyngeal stenosis as the radiation dose to the pharyngeal constrictor muscles was related to developing pharyngeal stenosis [2]. We speculated that the pathogenesis of Zenker diverticulum (ZD) might be related to the development of pharyngeal stenosis after CCRT. ZD is a diverticulum of the mucosa of the pharynx, just above the cricopharyngeal muscle. ZD is observed more often in the northern regions of Europe than in the southern regions; it is common in the United States, Canada, and Australia, but rare in Japan and Indonesia [19]. Although a complete understanding of the pathogenesis of ZD has not been reached despite a century of research [20], the most widely accepted theory is that the upper esophageal sphincter relaxation is inadequate resulting in incomplete opening of the upper esophageal sphincter and high intrabolus pressure. Histologically, the presence of inflammatory signals and development of fibrosis of the cricopharyngeal muscle have also been demonstrated [21]. Therefore, in Western people, the cricopharyngeal muscle is more likely to develop fibrosis and dilate incompletely at baseline, and is likely to become more fibromatic and not to dilate during and after CRT, resulting in pharyngeal stenosis, in the Western than in the Japanese population.

According to the report from Dana-Farber Cancer Institute, stricture, as evaluated by video swallow studies approximately 4–8 weeks after the end of the treatment, was observed in 36 (37%) of 96 patients who received IMRT with/without chemotherapy for head and neck cancer at various sites [22]. The primary site of the cancer was the oropharynx in 43, hypopharynx/larynx in 17, oral cavity in 13, nasopharynx in 11, maxillary sinus in 2, and unknown primary in 10 patients. The duration of feeding tube placement after RT completion was 0–3 months in 31(34%), 3–6 months for 26 (29%), 6–12 months for 23 (25%), and >1 year for 11 (12%) of the 91 patients requiring a feeding tube. However, the relation between stricture and G-tube dependence was not analyzed. We did not sequentially evaluate swallowing status by video swallow studies before, during, and after CCRT in this study, and we cannot find any paper reporting such an evaluation in Japanese patients. Stricture is considered to play an important role in the development of dysphagia, but it cannot, by itself, explain the condition. The pathogenetic mechanism of dysphagia after CCRT appears to be complicated. Various factors could be related to the development of dysphagia such as dry mouth, fibrosis of the neck, a decrease in sensation, edema, and so on. Therefore, we have to evaluate swallowing status sequentially before, during, and after CCRT using video swallowing studies in the near future.

As for differences in swallowing status between patients treated with IA- and IV-CCRT, Wakisaka *et al*. reported that the rate of patients with impaired oral intake at 6 months after therapy was 10% (4/40) in the IA arm, and 22.9% (8/35) in the IV arm [23]. According to the Dutch trial, the rate of those needing tube feeding was similar in both arms during the first 12 months [12]. However, 1/36 (2.8%) in the IA arm and 6/35 (17.1%) in the IV arm required tube feeding at the 5-year follow up [24]. In this study, G-tube use was seen one patient in each group. However, to draw any conclusions regarding which treatment is less likely to develop dysphagia, a larger number of cases is needed.

Recently, there has been greater emphasis placed on the importance of swallowing exercise. As mentioned above, Bhayani *et al*., from the MD Anderson Cancer Center, reported that one (3.4%) of 29 patients with HPC who continued follow-up remained dependent on a feeding tube at 2 years [18]. They concluded that adherence to an aggressive targeted swallowing exercise regimen may help to prevent long-term dependence on feeding tubes. We did not introduce swallowing exercise in this series. However, we introduced an opioid-based pain control program and support to allow patients to continue oral intake as far as possible during CCRT [25]. A staple of the Japanese diet is rice, and its stickiness and firmness can be varied during the cooking process. This might be helpful in the maintenance and early resumption of transoral feeding. Moreover, patients who receive CCRT are hospitalized during CCRT and generally discharged once the need for feeding tube support has ceased, similar to other patients in Japanese hospitals. This might lead staff to unconsciously provide prompt and adequate responses to patient condition. In addition, patients are also motivated to continue or resume transoral feeding by medical staff on a daily basis. These factors might have contributed to the prevention of dysphagia to some degree. As a result, 82 patients (86.3%) were able to receive oral intake at 1 month after CCRT in this study.

## Conclusions

This study suggests that Japanese patients are less likely to develop dysphagia early after CCRT than are Western patients. In particular, Japanese patients resume oral intake earlier than do Western patients. We speculated that the cricopharyngeal muscle condition in the Japanese population might differ from that in the Western population based on the fact that ZD is rare in Japan. Further study of the incidence of dysphagia after CCRT by ethnicity is required to clarify the differences in dysphagia after CCRT if an ethnic difference in the incidence of dysphagia after CCRT is confirmed.

## Supporting Information

S1 File The file contains all clinical data underlying the findings described in our manuscript. (XLSX)
